# Clinical Applications of Cancer-Associated Cells Present in the Blood of Cancer Patients

**DOI:** 10.3390/biomedicines10030587

**Published:** 2022-03-02

**Authors:** Cha-Mei Tang, Daniel L. Adams

**Affiliations:** 1 Creatv MicroTech, Inc., 9900 Belward Campus Drive, Suite 330, Rockville, MD 20850, USA; 2 Creatv MicroTech, Inc., 9 Deer Park Drive, Suite M5, Middlesex County, NJ 08852, USA; dan@creatvmicrotech.com

**Keywords:** cancer-associated macrophage-like cells CAMLs, CTCs, circulating tumor cells, immunotherapy, PD-L1, residual disease, companion diagnostics

## Abstract

The ability to obtain tumor material from cells in the blood of cancer patients provides a significant benefit over the use of tumor tissue as a diagnostic to make treatment decisions. However, the traditionally defined circulating tumor cell (CTC) has been shown to be useful only in some cases. A recently identified type of circulating stromal cell, which appears to be more frequent than CTCs, was found engulfing tumor material at the tumor site and then entering the blood stream. These cells were defined as cancer-associated macrophage-like cells (CAMLs). Together, CTCs and CAMLs may be able to provide information for cancer detection and diagnosis, without the use of tissue. CTCs and CAMLs have many clinical applications, three of which are summarized in this review: for prognosis, as companion diagnostics, and for residual disease monitoring.

## 1. Introduction

Cancer encompasses numerous subtypes and is the second leading cause of death in the United States. Worldwide, cancer deaths in 2020 were about 10 million [1]. Due to the variety of cancer subtypes, oncologists need a wide variety of diagnostics to help them make treatment decisions. Solutions to three such needs, indicated below, will be summarized in this paper. (1) Patients and oncologists want to know a patient’s prognosis at various times, such as at diagnosis, during therapy, and at the end of therapy. (2) Not every patient will benefit from a given drug, because many drugs are designed to target a specific tumor marker. The diagnostic to determine if a patient may benefit from a therapy is called companion diagnostic. Immunotherapy utilizes the patient’s own immune system to kill the tumor, but not all patients benefit from this therapy. Currently, all US FDA-approved companion diagnostics for immunotherapy are based on tissue [2,3,4,5]. However, a tissue biopsy can be expensive and sometimes carries a risk, such as pneumothorax, caused by tissue biopsy in lung cancer patients. (3) At the end of therapy, both the patient and the oncologist want to know if there is residual disease or if cancer has been totally eliminated. Imaging can aid in monitoring the growth of a tumor, but it requires time for a tumor to grow in size. A blood test that can determine residual disease before a tumor increases in size and without using radiation is desirable. A simple blood test may provide a solution to these issues.

Pathologically defined circulating tumor cells (CTCs) are actual tumor cells that have entered the blood stream [6,7,8,9]. When they are detected in the blood, they can be isolated and used for various clinical applications [10,11,12,13,14,15]. While CTCs have been readily found in metastatic breast, prostate, colorectal, and small cell lung (SCLC) cancer patients, they are rarely found in patients with non-metastatic carcinomas or with other solid tumors. Hence, the clinical applications of CTCs are limited.

In addition to CTCs, a circulating cell population that is a specific type of phagocytic cell has been identified in the tumor stroma. This particular type of stromal cell is a macrophage that engulfs tumor cells and debris within the tumor microenvironment and then enters the blood. These cells were named Cancer-Associated Macrophage-Like Cells (CAMLs), as they are CD14-positive and phagocytose tumor material [16,17,18,19,20,21,22,23,24,25,26,27,28,29,30]. CAMLs are very large, 25–300 µm in size, which makes it easy to distinguish them from CTCs and from normal monocytes in patients’ blood.

To date, CAMLs have been found in patients with 20 types of solid tumors, i.e., breast, esophageal, lung (NSCLC, SCLC, and other), prostate, pancreas (adenocarcinoma and acinar carcinoma), renal cell carcinoma (adeno and sarcomatoid), hepatocellular carcinoma, head and neck (numerous types), neuroblastoma (numerous types), sarcoma (numerous types), melanoma, neuroendocrine tumors, PNET, cholangiocarcinoma, ovarian, colon, uterine, urothelial, bladder, and endometrial tumors. Very likely CAMLs can be found in all solid tumors. CAMLs have also been found in cancers at all stages of development. However, CAMLs are not found in healthy individuals.

This review describes the type of information CTCs and CAMLs can provide to support prognosis, companion diagnostics, and monitoring of residual disease by a blood test.

Size-exclusion methods are ideal for isolating CTCs and CAMLs from the blood stream. The CellSieve^TM^ microfilter is a size-exclusion device with 7.5 µm-diameter pores, 180,000 pores distributed uniformly within a 9 mm-diameter area on a strong, low-autofluorescence, 10 µm-thick polymer. Images of the microfilter are shown in Reference [10].

Filtration by size is a suitable method to consistently capture multiple types of tumor-associated cells in the blood, both CTCs and CAMLs. Filtration can be performed under low pressure using a syringe pump or a vacuum pump [10].

For cell isolation, 7.5 mL of whole blood, collected in a CellSave Preservative Tube, is prefixed in 7.5 mL of prefixation buffer. The 15 mL sample is passed through the filter for 3 min. The microfilter removes all red blood cells and 99.9% of white blood cells [10,11,12]. The assay is followed by fixation, permeabilization, and fluorescence staining of the cells captured on the filter. The microfilter is then mounted on a glass slide [10,11,12] and can then be imaged on a fluorescent microscope.

**Figure 1:**
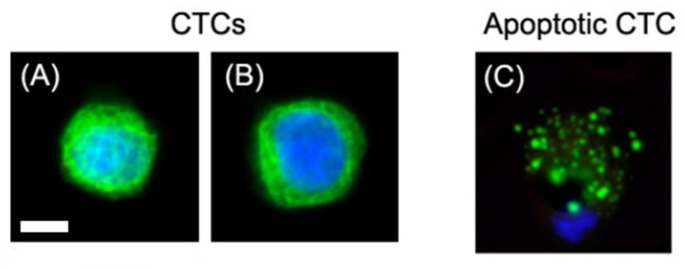
(**A**,**B**): Pathologically defined CTCs, where CK8, 18, 19 have a filamentous structure. CTCs do not express CD45. DAPI (blue), CK8, 18, 19 (green), and CD45 (violet). (**C**): Apoptotic CTC, where CK8, 18, 19 appear as dots. The scale bar is 10 µm.

Most invasive carcinomas are known to express cytokeratin filaments (CK) 8, 18, 19, while not expressing the white blood cell marker CD45. Typically, pathologically defined CTCs (PDCTCs) exhibit filamentous cytokeratin patterns. as shown in Figure 1A,B, but no CD45. When CTCs become apoptotic, cytokeratin collapses into blebs or dots, as shown in Figure 1C.

**Figure 2:**
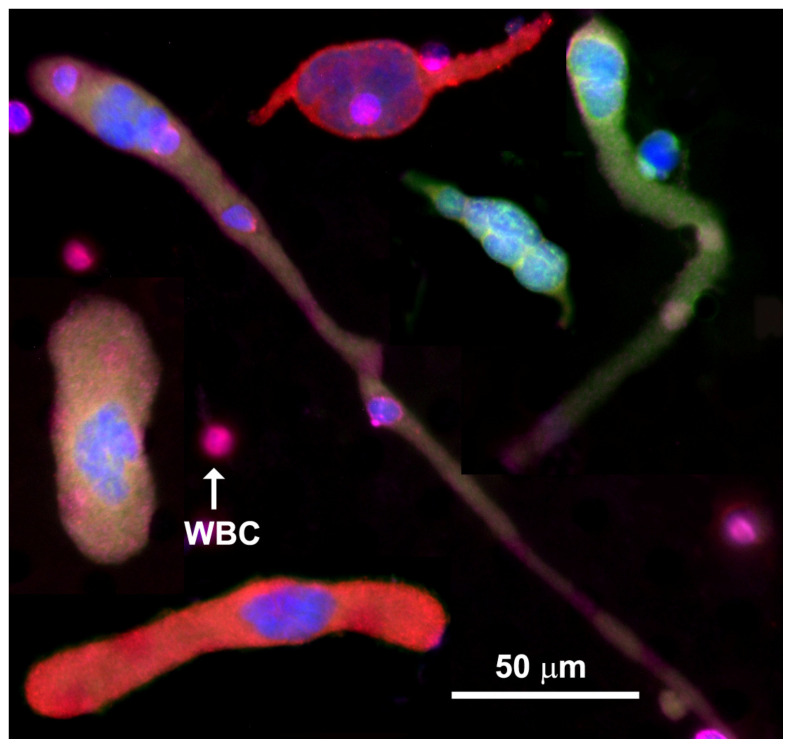
Even though every CAML has a different morphology, CAMLs have a few common features: a single tail or two tails on the opposite sides of the cell, and rod, oval or round shapes. CAMLs are polynucleated. DAPI (blue), CK8, 18, 19 (green), PD-L1 (red), and CD45 (violet). The scale is 50 µm.

CAMLs, shown in Figure 2, typically express CD14 and CD45, but contain diffuse cytokeratin. CAMLs have been found in a variety of shapes, yet have some universal common features, such as polynucleation and very large sizes, 25–300 µm. CAMLs are round or oval-shaped when small. As they grow larger, they can acquire a rod shape or grow one or two “tails” on opposite sides [16,17,18,19,20,21,22,23,24,25,26,27,28,29,30,31].

The low auto-fluorescence background of the filter material enables the determination of medium and low expression levels of the marker of interest and provides the ability to accurately measure CAML size.

Companion diagnostic assay development usually starts using cell lines that provide negative, low, medium, and high expression levels of the markers of interest and are screened against multiple antibodies for the marker of interest. Marker intensity on CAMLs and CTCs are validated using patient samples, and adjustment of reagents and expression levels is required to finalize the scaling criteria.

## 2. Prognosis

Both patients and oncologists are very interested in the prognosis of a patient’s disease. CAMLs can potentially provide broader prognostic information than CTCs. The number of CAMLs in a sample provides prognostic information, but the unusual property of CAML size provides even greater prognostic accuracy. In a presentation at the 2017 Annual ASCO meeting, 293 patients with six types of cancers—breast, prostate, pancreas, esophageal, lung, and kidney—were evaluated for 24 months [27]. The data found that patients with one or more CAML ≥ 50 µm had significantly shorter progression-free survival (PFS), with Hazard ratio of 3.7 (95% CI = 2.7−5.2, *p* < 0.001), and significantly shorter overall survival (OS), with Hazard ratio of 3.6 (95% CI = 2.5−5.5, *p* < 0.001), compared to patients with CAMLs <50 µm. 

For patients with pancreatic cancer, similar findings of PFS and OS were also shown in a paper published in 2021 [30]. The paper compared the use of CAML number, based on 12 CAMLs as the numerical cutoff, versus CAML size, based on 50 µm as the size cutoff. CAML number was not a significant indicator of OS, even though there was a trend toward a worse outcome for >12 CAMLs. For CAML size, it was found that patients with CAMLs ≥ 50 µm had PFS of 9.9 months, whereas patients with CAMLs < 50 µm did not reach mPFS in 24 months. Additionally, patients with CAMLs ≥ 50 µm had OS of 19.4 months, whereas patients with CAMLs < 50 µm did not reach mOS. This translated to patients with CAMLs ≥ 50 µm having significantly worse PFS (HR = 3.90, 95% CI 1.99–7.61, *p* < 0.001) and significantly worse OS (HR = 2.53, 95% CI 1.22–5.20, *p* = 0.019).

**Figure 3:**
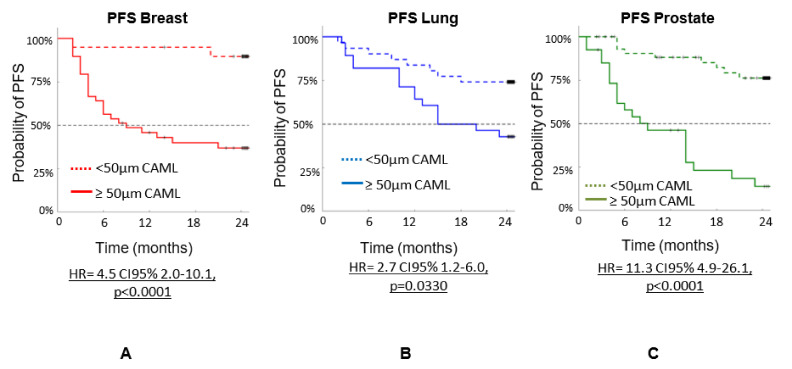
n = PFS of 192 patients with three types of cancers (breast, prostate, and lung). PFS is based on CAML size determined during a 24-month follow-up. (**A**) is PFS for breast cancer patients. (**B**) is PFS for lung cancer patients and (**C**) is PFS for prostate cancer patients. The data shows that patients with CAMLs ≥ 50 µm all has shorter PFS than patients with CAML < 50 µm independent of type of cancer.

In this review, we present data from 192 patients according to cancer, stage, and CAML size, including three different cancers: breast (n = 59), lung (n = 59), and prostate (n = 74), expending the number of patients in those three cancers from the 2017 ASCO presenation. This group comprised both treated and untreated patients. Figure 3 shows the progression-free survival (PFS) by cancer type using the 50 µm size as cutoff: (a) for breast cancer, n = 20 patients had CAMLs < 50 µm, and n = 39 patients had CAMLs ≥ 50 µm (HR = 4.5 CI95% 2.0–10.1, *p* < 0.0001), (b) for lung cancer, n = 31 patients had CAMLs < 50 µm, and n = 28 patients had CAMLs ≥ 50 µm (HR= 2.7 CI95% 1.2–6.0, *p* = 0.0330), and (c) for prostate cancer, n = 47 patients had CAMLs < 50 µm, and n = 27 patients had CAMLs ≥ 50 µm (HR = 11.3 CI95% 4.9–26.1, *p* < 0.0001).

**Figure 4:**
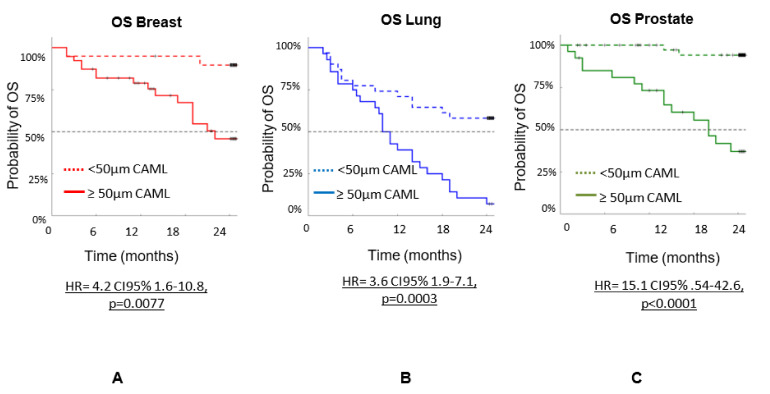
OS of n = 192 patients with three types of cancers (breast, prostate and lung). OS based on CAML size determined during a 24-month follow-up. (**A**) is OS for breast cancer patients. (**B**) is OS for lung cancer patients and (**C**) is OS for prostate cancer patients. The data shows that patients with CAMLs ≥ 50 µm all has shorter OS than patients with CAML < 50 µm independent of type of cancer.

Figure 4 shows the overall survival (OS) by cancer type using the 50 µm size as cutoff: (a) breast cancer (HR = 4.2 CI95% 1.6–10.8, *p* = 0.0077), (b) lung cancer (HR = 3.6 CI95% 1.9–7.1, *p* = 0.0003), and (c) prostate cancer (HR = 15.1 CI95%. 54–42.6, *p* < 0.0001). The data support that CAMLs are suitable for clinical applications involving patients with breast, lung, and prostate cancers.

**Figure 5:**
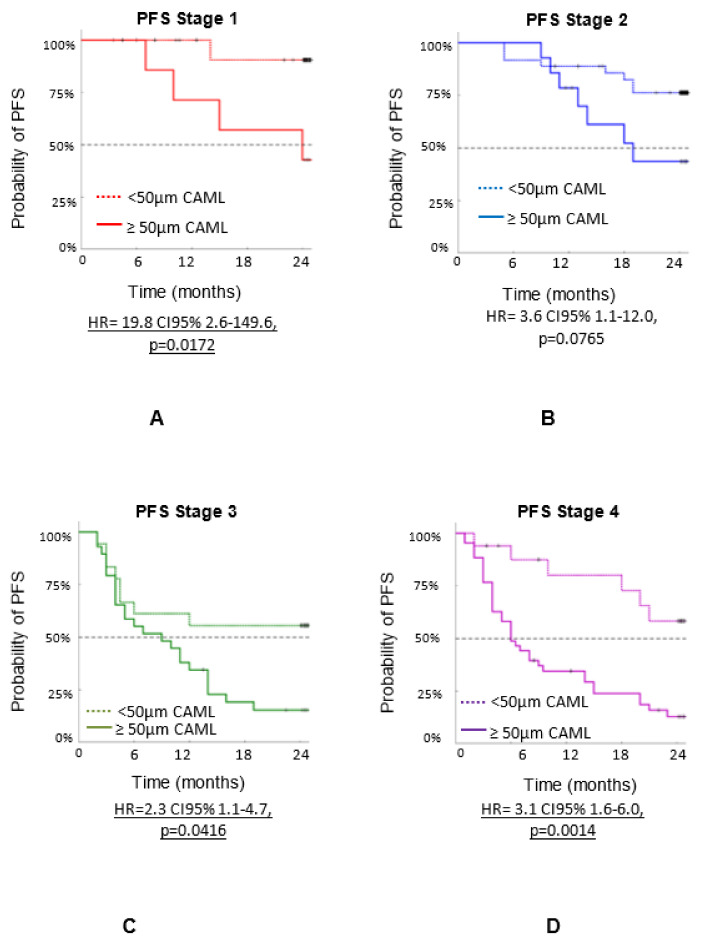
PFS of n = 192 patients with three types of cancers (breast, prostate, and lung) by stage. PFS based on CAML size at each stage of cancer, determined during a 24-month follow-up: (**A**) Stage 1, (**B**) Stage 2, (**C**) Stage 3 and (**D**) Stage 4.

Figure 5 shows the progression-free survival (PFS) by stage using the 50 µm size as cutoff in different stages: Stage 1 (n = 35), Stage 2 (n = 50), Stage 3 (n = 47), and Stage 4 (n = 60). (a) Stage 1, with (n = 27) patients having CAMLs < 50 µm, and (n = 8) with CAMLs ≥ 50 µm (HR = 19.8 CI95% 2.6–149.6, *p* = 0.0172), (b) Stage 2, with (n = 36) patients with CAMLs < 50 µm, and (n = 14) with CAMLs ≥50 µm (HR = 3.6 CI95% 1.1–12.0, *p* = 0.0765), (c) Stage 3, with (n = 18) patients with CAMLs < 50 µm, and (n = 29) with CAMLs ≥ 50 µm (HR = 2.3 CI95% 1.1–4.7, *p* = 0.0416), and (d) Stage 4, with (n = 17) patients with CAMLs < 50 µm, and (n = 43) with CAMLs ≥ 50 µm (HR = 3.1 CI95% 1.6–6.0, *p* = 0.0014).

**Figure 6:**
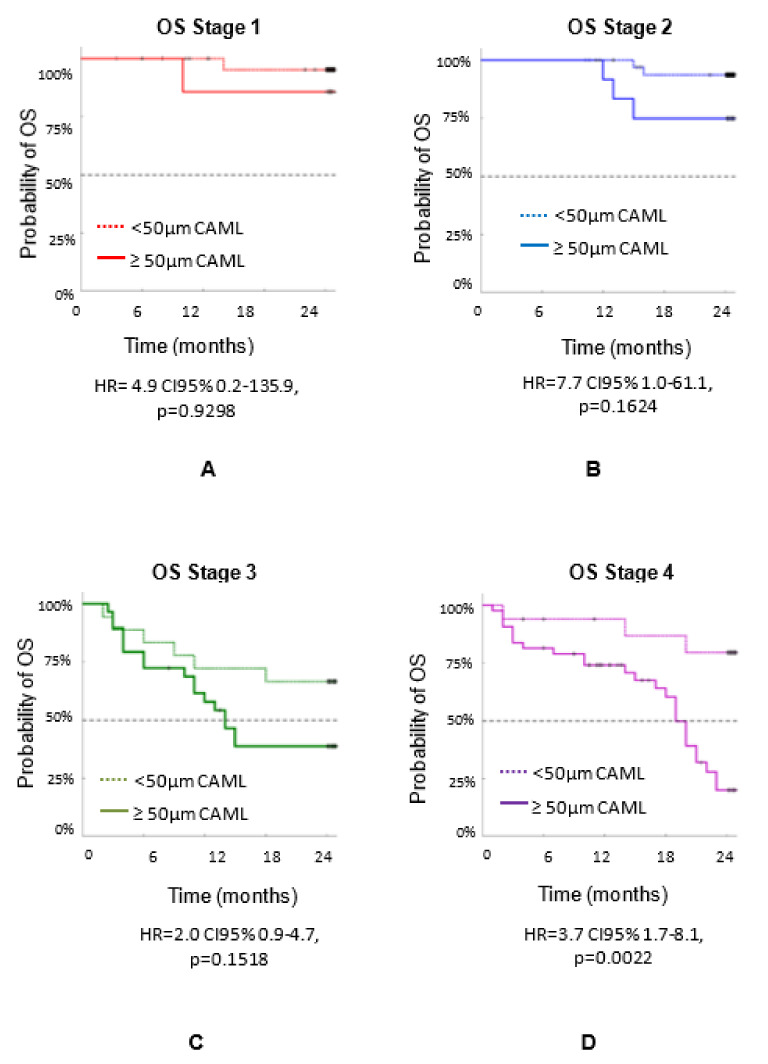
OS of n = 192 patients with three types of cancers (breast, prostate and lung) by stage. OS based on CAML size at each stage of cancer determined during a 24-month follow-up: (**A**) Stage 1, (**B**) Stage 2, (**C**) Stage 3 and (**D**) Stage 4.

Figure 6 shows the overall survival (OS) by stage using the 50 µm size as cutoff: (a) Stage 1 (HR = 4.9 CI95% 0.2–135.9, *p* = 0.9298), (b) Stage 2 (HR = 7.7 CI95% 1.0–61.1, *p* = 0.1624), (c) Stage 3 (HR = 2.0 CI95% 0.9–4.7, *p* = 0.1518), and (d) Stage 4 (HR = 3.7 CI95% 1.7–8.1, *p* = 0.0022).

**Figure 7:**
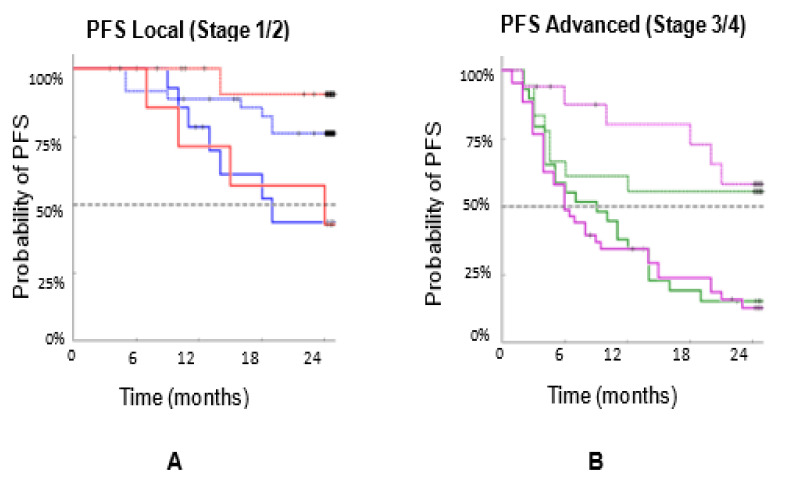
PFS of n = 192 patients with three types of cancers (breast, prostate, and lung) by stage. PFS based on CAML size, combining (**A**) Stages 1,2 (merging Figure 5A,B), and (**B**) Stages 3,4 (merging Figure 5C,D).

**Figure 8:**
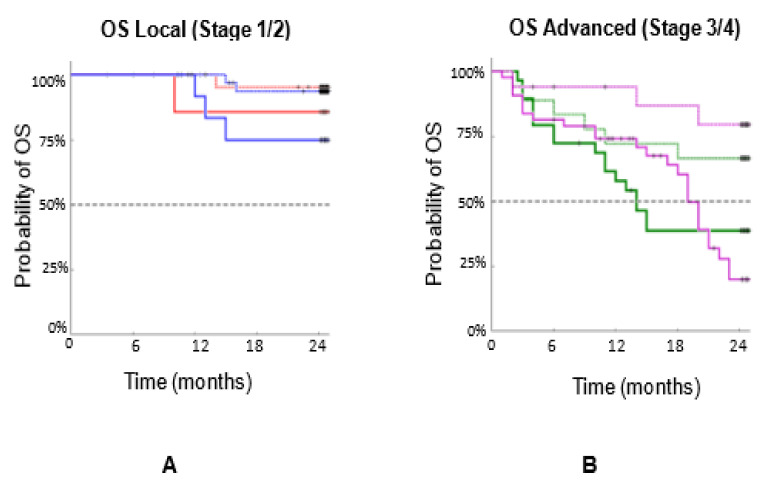
OS of n = 192 patients with three types of cancers (breast, prostate, and lung) by stage. OS based on CAML size, combining (**A**) Stages 1,2 (merging Figure 6A,B), and (**B**) Stages 3,4 (merging Figure 6C,D).

Figure 7A combines the PFS for Stages 1, 2, and Figure 7B combines PFS for Stages 3, 4. Figure 8A combines the OS for Stages 1, 2, and Figure 8B combines the OS for Stages 3, 4. These data support the hypothesis that large CAMLs are associated with more aggressive diseases. They also indicate that patients with cancer in more advanced stages have larger CAMLs.

CTCs are found primarily in patients with late-stage breast, prostate, colorectal cancers, and with small cell lung cancer. If one CTC is found in 7.5 mL of peripheral blood, then the prognosis is poor. An even worse prognosis is associated with the presence of one or more CTCs in mitosis [13].

**Figure 9:**
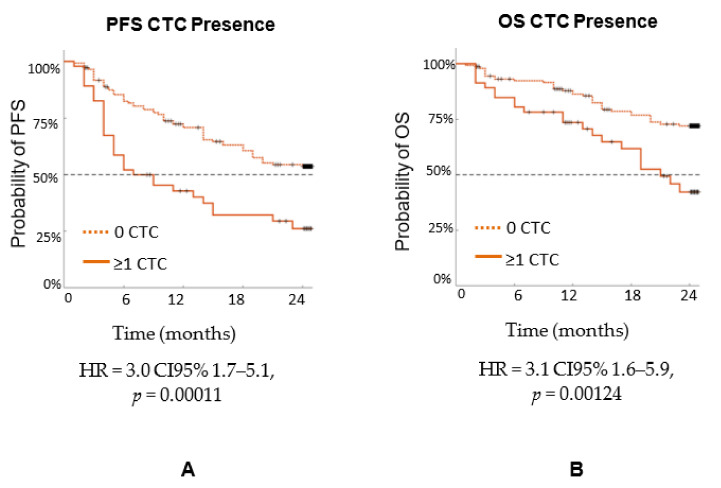
PFS of n = 192 patients with three types of cancers (breast, prostate, and lung) by stage (**A**) PFS and (**B**) OS based on the presence of CTCs, combining all stages.

**Figure 10:**
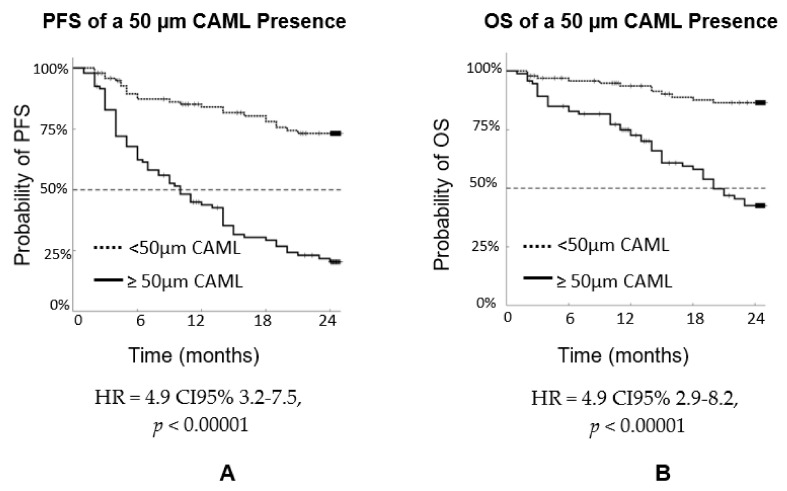
PFS of n = 192 patients with three types of cancers (breast, prostate, and lung) by stage (**A**) PFS and (**B**) OS based on CAML size, combining all stages.

The same 192 patients were analyzed for the presence of CTCs in relation to PFS and OS, combining all stages, as shown in Figure 9. For PFS, HR = 3.0 CI95% 1.7–5.1, *p* = 0.00011. For OS, HR = 3.1 CI95% 1.6–5.9, *p* = 0.00124. As the *p* values indicate, the presence of a single CTC indicated poor prognosis. Figure 10 analyzes the same 192 patients, combining all stages analyzed based on the presence of CAML size, using 50 µm as the cutoff. For PFS, HR = 4.9 CI95% 3.2–7.5, *p* < 0.00001. For OS, HR = 4.9 CI95% 2.9–8.2, *p* < 0.00001. As the *p* values indicate, CAML size is more informative than the CTC number.

## 3. Companion Diagnostics

Many therapies targeting markers on a tumor have been FDA-approved, and an even broader spectrum of promising therapies targeting different tumor markers are under development and in clinical trials. To determine the applicability of a particular therapy for a patient, the patient’s tumor must express the drug target at a meaningful level. A companion diagnostic is a clinical diagnostic test that matches a patient’s tumor drug target to a specific drug or therapy and is usually performed prior to intervention with the therapy.

The majority of companion diagnostics for the treatment of solid tumors rely on tumor tissue obtained by biopsy [2,3,4,5,6]. For many cancers, it is difficult and expensive to perform a biopsy. Furthermore, biopsies for some cancers can cause complications and carry risks to the health of the patient. For example, for lung cancer biopsies, a number of life-threatening events can occur, such as pneumothorax (i.e., a collapsed lung), bleeding in the lung cavity, or pneumonia. Pneumonia, or infection, is a risk for all types of lung biopsies and is caused by the introduction of bacteria or other foreign material during the biopsy. Pneumothorax, where air leaks out between the lung and chest cavity, can cause difficulty breathing or cause the lung to collapse, necessitating invasive chest tube placement and hospitalization.

Recently, a number of blood-based companion diagnostic tests and complimentary diagnostic tests have been approved by the FDA, such as the FoundationOne Liquid CDx and Guardant 360 CDx tests. However, these diagnostics are only applicable for highly specific genetic changes found in cell-free DNA in patient blood [3,4,5]. Their use is limited, because they cannot determine the expression of tumor markers, such as PD-L1 for immunotherapies.

Cell-based liquid biopsy can deliver companion diagnostics for therapy markers as well as mutations of the cancer. The assay can deliver tumor DNA in the CTCs and CAMLs for sequencing for genetic information, though sequencing will not be covered in this review paper. The method for providing companion diagnostics for drug targets on the surface of tumor cells is described below.

Typically, three fluorescent channels are adequate to identify CTCs and CAMLs. There is at least one additional fluorescent channel available for use to measure a drug marker.

We have developed assays for more than 20 drug targets on CTCs and CAMLs, such as PD-L1, PD-L2, CCR5, HLA-DRB3, CXCR4, HER2, p-ERK, PRAME, HHLA2, FGFR1, FGFR2, FGFR3, and others. Clinical validations backed by a large number of patients with a variety of cancers are important and are on-going.

For carcinomas, cytokeratins are used to identify CTCs. For sarcomas, vimentin is used to identify CTCs, as sarcomas are not epithelial in origin and thus do not express cytokeratins [24].

We describe two liquid biopsy companion diagnostic examples, PD-L1 and CCR5, which illustrate how the expression of these markers and their variation provides useful clinical information.

### 3.1. Companion Diagnostic for PD-L1

PD-L1, expressed on the surface of tumor cells, suppresses immune recognition by binding to its receptor PD-1, found on activated T cells, B cells, and myeloid cells. Blocking the PD-L1–PD-1 axis with a number of FDA-approved cancer immunotherapies activates the immune system to unleash CD8 T cells to kill the tumor. High PD-L1 expression on tumor cells is desirable for immunotherapy [32,33,34,35]. The relative expression of PD-L1 in tumors is well regarded as a predictor of response to these immunotherapies utilizing tissue-based companion diagnostics.

Currently, five immunotherapy drugs have received multiple FDA approvals for a wide spectrum of cancer therapies: Opdivo^®^ (nivolumab) of BMS, Keytruda^®^ (pembrolizumab) of Merck, Tecentriq^®^ (atezolizumab) of Genentech, Imfinzi (durvalumab) of AstraZeneca, and Libtayo^®^ (cemiplimab-rwlc) of Regeneron and Senofi-Aventis. All three FDA-approved companion diagnostics for those immunotherapies are based on tissue: Dako^®^ PD-L1 IHC 22C3 pharmDx, Dako^®^ PD-L1 IHC 28-8 pharmDx and Ventana^®^ PD-L1 SP142 [2,3,4,5].

**Figure 11:**
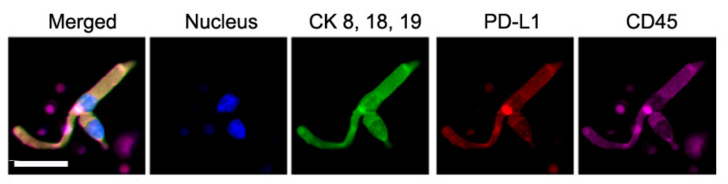
Image of two CAMLs next to each other. CAMLs were stained for CK8, 18, 19, PD-L1, and CD45, which appeared in the cytoplasm. The scale bar is 50 µm.

While PD-L1 is detected on the surface of tissue and CTCs, it is also found inside CAMLs, because CAMLs engulf tumor cells and tumor debris. PD-L1 is in CAML cytoplasm. Figure 11 shows two CAMLs, one large and one small, next to each other. In both CAMLs, cytokeratins, PD-L1, and CD45 are almost uniformly distributed in the cytoplasm.

**Figure 12:**
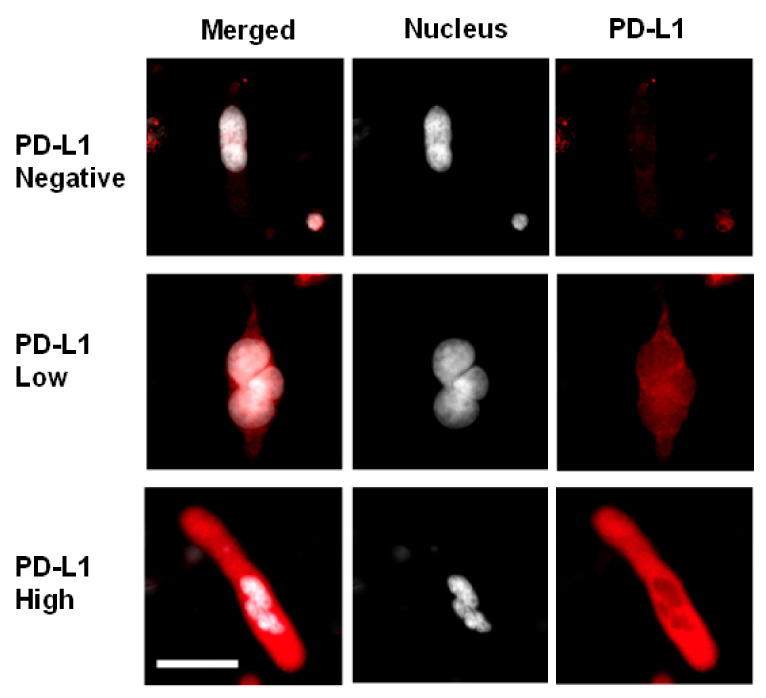
PD-L1 expression is determined in CAMLs by the intensity of PD-L1 staining by microscope imaging: PD-L1 (red), Nucleus (white). The cytokeratins and CD45 channels are not displayed. The scoring is based on the average value of PD-L1 staining in the CAML. The white color is used for the nucleus to make the merged image of PD-L1 negative and low-expression cells more visible. The scale bar is 50 µm.

Figure 12 shows three different PD-L1 expression levels in CAMLs: negative, low, and high. This PD-L1 expression scoring is based on the intensity of the PD-L1 staining obtained by microscope observation. To make the PD-L1 more visible, the nucleus is shown in white. PD-L1 was not detected inside the nucleus area.

Chemoradiation therapy can change PD-L1 expression, which can enhance the response of some patients who receive subsequent immunotherapy. For those patients, the best time to perform PD-L1 companion diagnostics for immunotherapy is after chemoradiation therapy [29].

The PD-L1 assay has been extensively validated for many types of cancers in more than 2000 patient samples, especially for non-small cell lung cancer (NSCLC) and breast cancer. The data include patients treated with four different immunotherapy drugs (nivolumab, pembrolizumab, atezolizumab, and durvalumab), as well as patients treated with immunotherapy–drug combinations.

### 3.2. Companion Diagnostic for CCR5

CCR5, also known as C–C chemokine receptor type 5, is predominantly expressed on T cells, macrophages, dendritic cells, eosinophils, microglia, and cancer cells. Approximately 50% of human breast cancers express CCR5. CCR5 has been found to be upregulated in aggressive breast cancer, especially in triple-negative breast cancer. CCR5 can also be expressed on a variety of other solid tumors.

CCR5 usually appears in pools or as small dots on the surface of tumor cells, when CCR5 becomes activated by chemokine C–C Ligand 5 (CCL5) (e.g., RANTES) [32]. Activated CCR5 pools are endocytosed and then translocated to the perinuclear space. Once in the perinuclear space, RANTES is degraded. CCR5 receptors are re-sensitized and recycled back to the cell surface.

**Figure 13:**
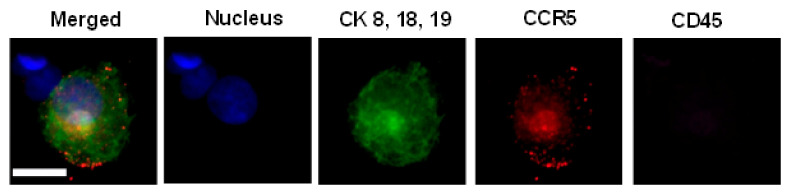
CCR5 expression appears as dots on the surface of and inside this CTC. The scale bar is 15 µm.

**Figure 14:**
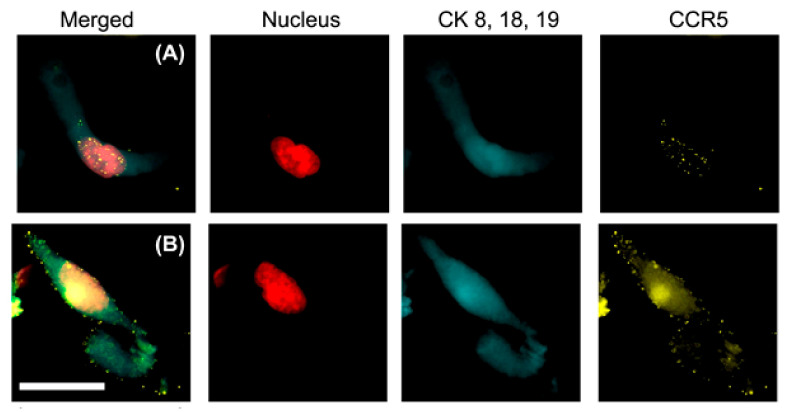
(**A**) CCR5 appears as dots in the cytoplasm of the CAMLs and migrates to the nucleus of the CAML. (**B**) The CCR5 dots then migrate to the surface. The scale bar is 50 µm.

CCR5 appears in pools on the surface of CTCs, as shown in Figure 13. After CAMLs have engulfed tumor cells, CCR5 appears in pools inside the cytoplasm and enters the nucleus, as shown in Figure 14A. After RANTES is degraded, CCR5 pools migrate to the surface of CAMLs, as shown in Figure 14B. The colors of nucleus, cytoplasm, and CCR5 are modified in Figure 14 to make the CCR5 pools more visible in the CAMLs.

The determination of CCR5 expression in CTCs and CAMLs is very different from that of PD-L1 expression. For CCR5, clinical data show that the aggressiveness of a cancer is related to the number of CCR5 dots. A manuscript has been submitted for publication on this phenomenon [36]. Currently, the CCR5 assay is very useful for both drug development and basic research on CCR5.

### 3.3. Summary of Companion Diagnostic Application

Since CAMLs are easy to identify by their shape and features, it is possible to eliminate CD45 in the assay to allow two or more targeted markers in the same assay. It is also possible to first stain for cytokeratin and C45 to identify the cells and then quench the cytokeratin and CD45 after imaging, so that the cells can be re-stained for three different drug targets. The quenching technique can be repeated to analyze 12–15 markers on the same cells [26]. With advances in microscopes and fluorescent dyes, it should be possible to analyze even more markers simultaneously in the future.

As observed in tissue biopsies, the expression of the marker of interest is variable among the cells. Similarly, for CAMLs, a marker expression is not uniform in all the CAMLs in a blood sample. The scoring also varies across different tumor types. The establishment of a scoring indicating high, medium, and low expression of the marker needs to be validated by analyzing a sufficient number of patient samples for each type of cancer, with known patient clinical information.

Companion diagnostics based on cells captured on the microfilter can be performed at desirable time points and can be easily repeated. This flexibility is useful, because some treatments can affect the expression of markers over time.

## 4. Residual Disease

Treatment is necessary to improve the survival of cancer patients. However, oncologists have few options to determine if a tumor has been eradicated at the end of standard therapy. For example, a surgeon may not know if surgery has removed all of the malignant tissue. Chemotherapy is frequently administered after surgery to reduce the chance of residual disease. The existence of residual disease is commonly regarded as an indicator of poor prognosis in all cancers. 

Initial studies on tracking CAMLs have found that the number and size of CAMLs at the end of treatment confirm the existence of residual disease, as well as its aggressiveness, as described earlier.

**Figure 15:**
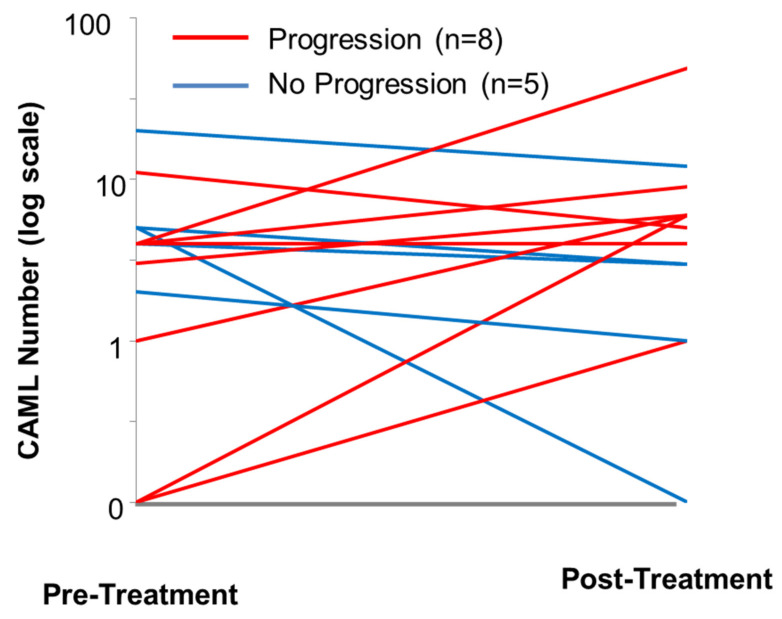
CAML enumeration in patients who had pancreatic surgery to determine whether the patients will likely progress within 2 years versus CAML enumeration in patients who did not progress. Blood was collected at pre-treatment and post-treatment time points. Among patients who experienced progression during treatment (red lines), six patients presented an increase in CAML number, one patient showed equal numbers, and one a decrease in CAML number from 11 to 5 CAMLs. All five patients who experienced no progression during treatment (blue lines) presented a decrease in CAML number.

An example is given in Figure 15, based on the change of CAML number before and after surgery for pancreatic cancer. The data were presented in a publication by Gardner et al., in the Nature Partner Journal (npj) *Precision Oncology* in 2021 [31]. Though these are preliminary pilot findings, it was reported that n = 6/8 patients who experienced progression showed an increase in CAML number in their post-surgical blood samples, one patient had the same number of CAMLs, and one patient presented a decrease from 11 to 5 CAMLs. By contrast, all patients (n = 5) who showed a decrease in CAML number experienced no progression during a 2-year period (blue lines). Similar patterns were not observed while tracking CA19-9 or CEA [31].

## 5. Discussion

In summary, prognosis, companion diagnostics, and residual disease determination by blood test would be beneficial to patients and oncologists. CAMLs in combination with CTCs as a liquid biopsy provide a source of tumor material from the patient. CTCs are found in patients with SCLC and late-stage breast, prostate, and colorectal cancers, while CAMLs are consistently found in patients with all 20 types of solid tumors analyzed and in all stages of disease progression; therefore, CAMLs are able to provide reliable tumor material. In addition, CAML size provides very useful information in addition to CAML number.

We presented data from many studies of PFS and OS of patients followed up for 24 months (1) combining patients with breast, prostate, pancreas, esophageal, lung, and kidney cancers, (2) breast, prostate, and lung cancers analyzed as individual cancers, (3) analyzing the use of CTC number versus CAML size, and (4) analyzing the effect of cancer stage. In summary, the common conclusion is that the presence of one CAML ≥ 50 µm in 7.5 mL of whole blood is a predictor of poor prognosis.

Many cancer drugs are under development to target specific markers expressed by tumor cells on their surface. The response of the patient to the therapy depends on whether the patient’s tumor expresses those markers. CTCs can be used to develop companion diagnostics, but the problem of solely depending on CTCs is that they are not always present in 7.5 mL of whole blood. This paper showed that CAMLs can be used to determine whether a patient will benefit from a given drug. The drug target can behave very differently. PD-L1 marker staining is diffuse inside CAMLs and can be helpful to determine the response to immunotherapy. The intensity of PD-L1 fluorescent staining in CAMLs provides information about PD-L1 expression. The CCR5 marker, however, appears as dots inside or on the surface of the CAMLs. The level of CCR5 expression is based on the number of CCR5 dots. Companion or complementary diagnostics can be developed by a blood test utilizing markers in CAMLs.

At the end of therapy, the patient and the oncologist need to know if the patient still has cancer as well as the aggressiveness of the disease. Again, CAMLs can provide this information through a comparison of CAML number and size before and at the termination of therapy. An example is given in this review, showing that the decrease of CAML number at the end of therapy correlated with longer PFS.

Cell-based liquid biopsies have broad capabilities beyond those described in this paper based on our clinical data; they can: (a) predict patients’ response to a new therapy after the initial treatment, (b) monitor the response to therapy over time, (c) detect cancer recurrence in patients in remission, (d) allow cancer screening for a single cancer or multiple cancers, and (e) deliver whole-tumor DNA for sequencing.

Liquid biopsies combining CTCs and CAMLs can also provide approaches for basic research on cancer and cancer biomarkers not requiring tissue and for drug development.

## Data Availability

All datasets used and/or analyzed throughout this study are available from the corresponding author based on sensible request.

## Abbreviations

CAMLs	Cancer-Associated Macrophage-like Cells (CAMLs)
CTCs	Circulating Tumor Cells
NSCLC	Non-Small Cell Lung Cancer
SCLC	Small Cell Lung Cancer
PD-L1	programed death Ligand 1
CCR5	C–C chemokine receptor type 5
